# The Urgent Need for Novel Antimicrobial Agents and Strategies to Fight Antibiotic Resistance

**DOI:** 10.3390/antibiotics8040254

**Published:** 2019-12-06

**Authors:** Marco Maria D’Andrea, Maurizio Fraziano, Maria Cristina Thaller, Gian Maria Rossolini

**Affiliations:** 1 Department of Biology, University of Rome “Tor Vergata”, 00133 Rome, Italy; fraziano@bio.uniroma2.it (M.F.); thaller@uniroma2.it (M.C.T.); 2 Department of Experimental and Clinical Medicine, University of Florence, 50134 Florence, Italy; gianmaria.rossolini@unifi.it; 3 Clinical Microbiology and Virology Unit, Florence Careggi University Hospital, 50134 Florence, Italy

**Keywords:** antibiotics, infections, novel antimicrobial approaches

Antibiotic resistance in bacterial pathogens has currently reached very high and alarming levels. Indeed, according to a number of international bodies, this “antibiotic resistance crisis” could possibly bring us back to a “pre-antibiotic era” in the near future, if no effective actions are promptly undertaken [1,2]. Unfortunately, the slow progress in antibiotic discovery and development observed during the last decades has led to a limited availability of new compounds for clinical use, thus exacerbating the burden of antibiotic resistance on morbidity and mortality rates. In this scenario, also many modern clinical practices associated with an increased risk of infection and in which antibiotics are essential (e.g., anti-cancer treatments, solid organ and stem cell transplantations, or implantation of prosthetic devices) are seriously at risk of success [3].

In this perspective, it is clear that there is an urgent need to discover and develop novel antibiotics, as well as to develop innovative antimicrobial approaches, possibly acting in synergy with standard chemotherapy. Indeed, even if the interest in the field of antibiotic research and development is recovering, there are still several unmet clinical needs that wait to be addressed in a timely manner.

This Special Issue of *Antibiotics* collected several contributions showing interesting descriptions of novel molecules with promising antimicrobial activities, as well as alternative approaches to fight bacterial infections and the spread of resistant bacteria. Specifically, Nunvar et al. investigated the mechanisms of action and the resistance mechanism of a recently discovered synthetic 2-thiocyanatopyridine derivative, which was previously found to have antibacterial activity against *Mycobacterium tuberculosis* and *Burkholderia cenocepacia* strains. The work nicely demonstrated that the growth inhibitory effect of this compound is linked to a repression of the translation machinery and also elucidated that resistance is related to the over-expression of two Resistance Nodulation Division (RND)-type efflux-pumps [4]. Another paper described the growth inhibitory effects of several antimicrobial peptides belonging to the first- and second-generation ceragenin family against clinical isolates of multi-drug resistant (MDR) *Klebsiella pneumoniae*. Interestingly, the mechanisms leading to resistance to another widely used antimicrobial peptide, i.e., colistin, did not impact the activity of this class of molecules, which could therefore represent very attractive lead compounds to be further developed for the treatment of infections caused by MDR strains of *K. pneumoniae* [5]. Patil et al. identified a new potential lead drug candidate which exhibited remarkable growth inhibitory activity towards several opportunistic pathogens, in particular, *Acinetobacter baumannii*. This adamantyl urea derivative, selected after a screening of 17 new similar derivatives, targets the penicillin-binding protein 1a of this species and represents an excellent candidate to be used as lead molecule for the rationale design of similar compounds [6]. Other interesting lead molecules, tested against isolates of *Staphylococcus aureus* and *Enterococcus* spp. and belonging to two different thieno[*b*]carbazole classes, were obtained by Seethaler et al. [7]. The authors of this work tested the antimicrobial activity of 21 of these compounds and also evaluated the protection ability of selected derivatives in an in vivo *Galleria mellonella* infection model, showing promising results for at least one of these compounds [7]. Lastly, Antonelli et al. characterized the in vitro antimicrobial activity towards different bacterial and fungal pathogens of a commercial desiccating agent, which was previously demonstrated to be effective for the treatment of dental biofilms. The work demonstrated a broad-spectrum antibacterial and antifungal activity of this product, that it is not affected by resistance to classical antibiotics and therefore might be exploited for the treatment of periodontitis, endodontic infections, and ulcers [8].

Other contributions showed a remarkable antimicrobial effect, alone or in synergy with standard antibiotics, of some well-known agents that are generally regarded as non-toxic, such as lactic acid, N-acetylcysteine, and chlorophyllin [9,10,11], thus providing very interesting examples of possible drug repositioning. Specifically, Bardhan and colleagues reported on the antimicrobial activity of lactic acid, a natural product often present in naturally fermented substances, against MDR and carbapenem-resistant clinical strains of *K. pneumoniae*. This work demonstrated the prominent ability of this compound to reduce the bacterial load of both planktonic cells and biofilms produced by these strains, thus highlighting the potential of lactic acid as a cost-effective antimicrobial and disinfectant for the effective removal of biofilms [9]. Ciacci et al. demonstrated that N-acetylcysteine, in combination with colistin at concentrations likely achievable by topical administration, might represents a valid alternative for the treatment of respiratory infections caused by *Stenotrophomonas maltophilia*. Results from this study are very exciting, given the major role of *S. maltophilia* in difficult-to-treat respiratory tract infections in cystic fibrosis patients and, at the same time, the possibility to administer these two compounds via inhalation, likely reaching concentrations above the minimum inhibitory concentration (MIC) [10]. On the other hand, Richter et al. showed the growth inhibitory effect of colistin plus chlorophyllin against *Escherichia coli* and *Salmonella typhimurium*. This combination, which is active also against colistin-resistant *E. coli* strains carrying the *mcr-1* transferable resistance gene, suggests an interesting application of this combination for the treatment of infections by drug-resistant strains [11].

The renewed interest in the use of bacteriophages or components thereof in the battle against pathogenic bacteria is documented by the paper by Swift et al. that characterized an endopeptidase encoded by a prophage located in the genome of a *Bacillus cereus* strain, whose gene was detected by using bioinformatics screening. This work showed the very promising lytic activity of this endopeptidase that was selectively active against all *Bacillus* spp. tested strains, thus representing a very interesting tool for the treatment of infections sustained by members of these species [12].

Lastly, the Special Issue includes also an interesting meta-analysis on the efficacy and safety of tedizolid, in comparison to linezolid, for the treatment of acute bacterial skin and skin structure infections. Results from this work showed that tedizolid was non-inferior in efficacy for the treatment of this type of infections, was generally as well-tolerated as linezolid, and had an incidence of important adverse effects lower than that of linezolid, thus contributing to the knowledge about the efficacy and safety of these drugs [13].

In conclusion, different novel and heterogeneous approaches for the fight against antibiotic resistant bacteria have been collected in this Special Issue. According to the main message from the report of the World Health Organization to the secretary-general of the United Nations, there is “no time to wait” [2], and antimicrobial resistance must be addressed by concerted actions deployed on several fronts. The discovery and development of novel classes of antibiotics, the modifications or the repositioning of existing drugs, and the identification of novel antimicrobial therapies are all promising approaches, not mutually exclusive, to fight antibiotic resistance. These actions need a coordinated and multidisciplinary effort which has to be stimulated and funded by specific programs undertaken at an international level.

## References

[1] O’Neill J. (2016). Tackling Drug-Resistant Infections Globally: Final Report and Recommendations. The Review on Antimicrobial Resistance. https://amr-review.org/sites/default/files/160518_Final%20paper_with%20cover.pdf.

[2] Interagency Coordination Group on Antimicrobial Resistance (2019). No Time to Wait: Securing the Future from Drug-Resistant Infections Report to the Secretary-General of the United Nations. https://www.who.int/antimicrobial-resistance/interagency-coordination-group/IACG_final_report_EN.pdf?ua=1.

[3] Hutchings M., Truman A., Wilkinson B. (2019). Antibiotics: Past, present and future. Curr. Opin. Microbiol..

[4] Nunvar J., Hogan A.M., Buroni S., Savina S., Makarov V., Cardona S.T., Drevinek P. (2019). The effect of 2-thiocyanatopyridine derivative 11026103 on *Burkholderia cenocepacia*: Resistance mechanisms and systemic impact. Antibiotics.

[5] Ozbek-Celik B., Damar-Celik D., Mataraci-Kara E., Bozkurt-Guzel C., Savage P.B. (2019). Comparative in vitro activities of first and second-generation ceragenins alone and in combination with antibiotics against multidrug-resistant *Klebsiella pneumoniae* strains. Antibiotics.

[6] Patil M., Noonikara-Poyil A., Joshi S.D., Patil S.A., Patil S.A., Bugarin A. (2019). New urea derivatives as potential antimicrobial agents: Synthesis, biological evaluation, and molecular docking studies. Antibiotics.

[7] Seethaler M., Hertlein T., Wecklein B., Ymeraj A., Ohlsen K., Lalk M., Hilgeroth A. (2019). Novel small-molecule antibacterials against Gram-positive pathogens of *Staphylococcus* and *Enterococcus* species. Antibiotics.

[8] Antonelli A., Giovannini L., Baccani I., Giuliani V., Pace R., Rossolini G.M. (2019). In vitro antimicrobial activity of the decontaminant HybenX^®^ compared to chlorhexidine and sodium hypochlorite against common bacterial and yeast pathogens. Antibiotics.

[9] Bardhan T., Chakraborty M., Bhattacharjee B. (2019). Bactericidal activity of lactic acid against clinical, carbapenem-hydrolyzing, multi-drug-resistant *Klebsiella pneumoniae* planktonic and biofilm-forming cells. Antibiotics.

[10] Ciacci N., Boncompagni S., Valzano F., Cariani L., Aliberti S., Blasi F., Pollini S., Rossolini G.M., Pallecchi L. (2019). In vitro synergism of colistin and N-acetylcysteine against *Stenotrophomonas maltophilia*. Antibiotics.

[11] Richter P., Krüger M., Prasad B., Gastiger S., Bodenschatz M., Wieder F., Burkovski A., Geißdörfer W., Lebert M., Strauch S.M. (2019). Using colistin as a trojan horse: Inactivation of Gram-negative bacteria with chlorophyllin. Antibiotics.

[12] Swift S.M., Etobayeva I.V., Reid K.P., Waters J.J., Oakley B.B., Donovan D.M., Nelson D.C. (2019). Characterization of LysBC17, a lytic endopeptidase from *Bacillus cereus*. Antibiotics.

[13] Lan S.-H., Lin W.-T., Chang S.-P., Lu L.-C., Chao C.-M., Lai C.-C., Wang J.-H. (2019). Tedizolid versus linezolid for the treatment of acute bacterial skin and skin structure infection: A systematic review and meta-analysis. Antibiotics.

